# Supplementary material for: Characterization and engineering of the type 3 secretion system needle monomer from Salmonella through the construction and screening of a comprehensive mutagenesis library
Source: mSphere. 2024 Aug 7;9(8):e00367-24. doi: 10.1128/msphere.00367-24 (PMC11351046; doi:10.1128/msphere.00367-24)
Supplement: Supplemental material — Figures S1 and S2, supplemental methods, and Tables S1 to S6. [file msphere.00367-24-s0001.docx]

**Supplementary Data**

**
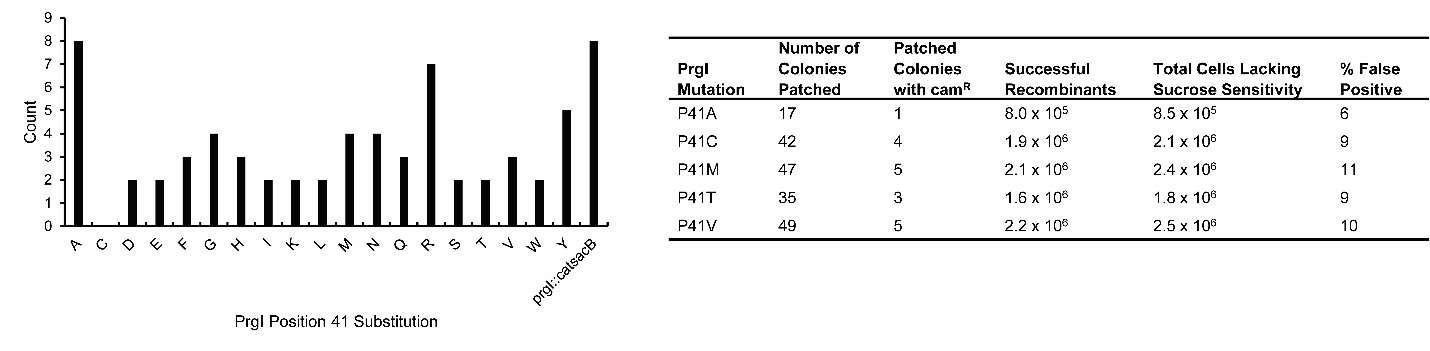
**

**Supplementary Figure 1 – Recombineering results for the PrgI^P41^ library.** An equimolar pool of all 20 fragments of the saturation mutagenesis library was introduced into an *sptP::sptP^1-167^-phoA prgI::catG-sacB* strain using a single λ Red recombineering event, and 68 of the resulting clones were Sanger sequenced. On the left is the count of each PrgI allele in the 68 clones. On the right are the results of conducting 5 separate recombineering events to produce individual PrgI alleles.


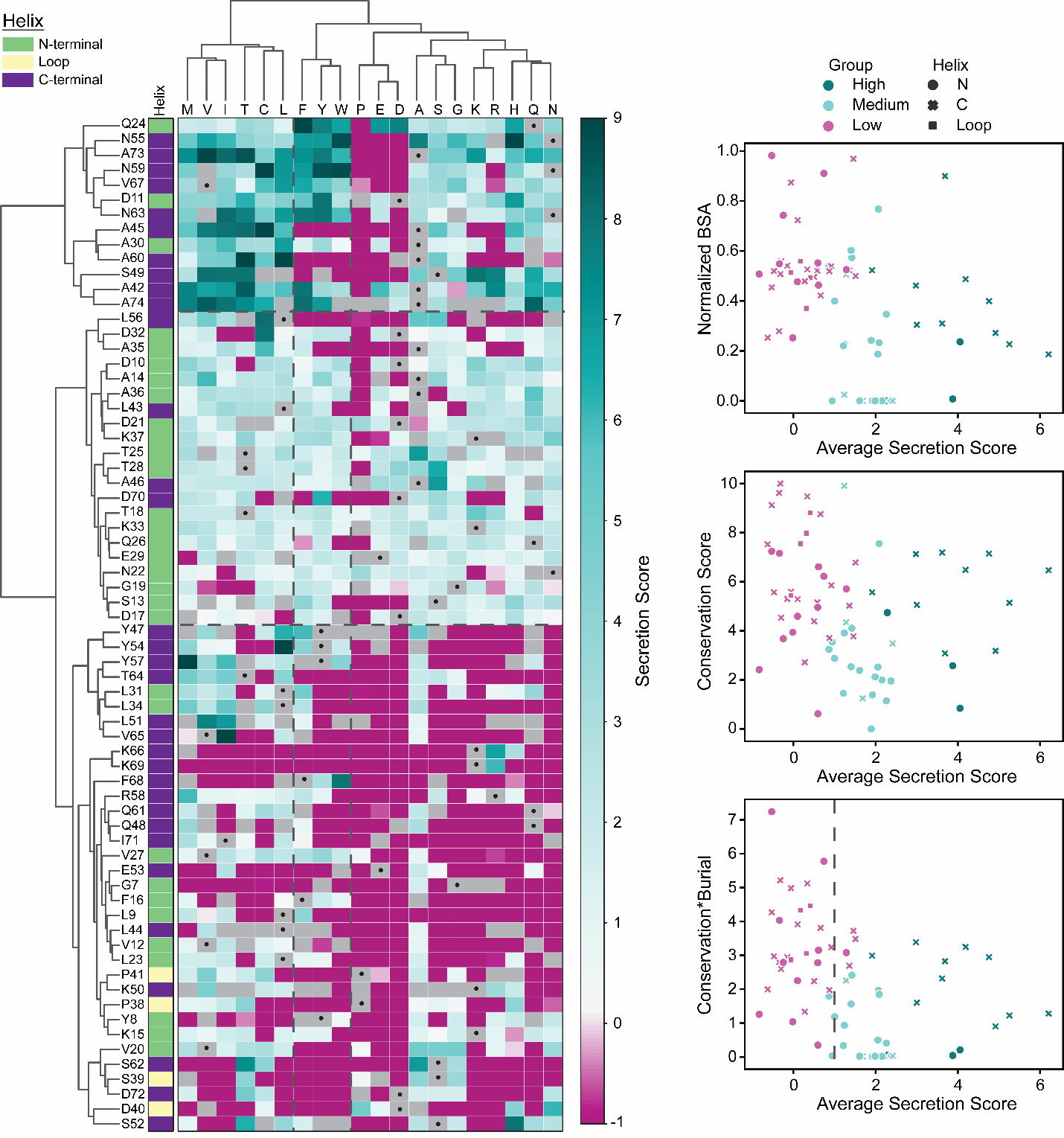


**Supplementary Figure 2 – Clustering patterns of the PrgI SFL.** Structural elements tended to cluster together (left), with the N-terminal helix being generally mutationally tolerant but not producing many high secreting variants. The highest secreting variants (top left cluster, dark blue) were surprisingly located in the interior N-terminal helix and were often large hydrophobic residues. Trends in the ability of BSA and conservation to predict secretion score are shown on the right.

**Supplementary Methods**

*Sample preparation for high-throughput sequencing*

The randomly arrayed glycerol stocks were inoculated in identical arrangements in fresh media in sterile, flat-bottom 96-well plates using a Tecan Fluent and grown with lids in DigiLab HiGro shaking stacks at 37°C, 200 rpm for 18 hours. Each clone was assigned a pool according to its relative secretion titer. A Tecan Fluent was used to reformat clones and sort them into their assigned pools. A VBA macro assigned pools according to relative secretion titer and assigned clones to a new plate and well ID to provide instructions for the Tecan Fluent. 150 μL of the fresh cell suspension was mixed with 50 μL 60% glycerol in a fresh sterile, round-bottom 96-well plate (Corning). The Tecan Fluent failed after sorting pools F-J, so the remainder were done by hand over the course of a week. Each well of the newly sorted glycerol stocks was sampled and pooled according to **Table S4** for genomic DNA purification. Genomic DNA was purified from 1 mL of each pool and 0.5 mL of the naïve library using the GenElute Bacterial Genomic DNA kit (Sigma).

PCR for library preparation was conducted with Phusion polymerase. The purified genomes from the pool mixtures were amplified using the “Round 1” reaction recipe (**Table S5**) and cycling conditions (**Table S6**) with primers oLAB278 and oLAB279 (**Table S3**) to attach Illumina Nextera XT adapters. Reactions were purified using the Promega Wizard SV PCR cleanup kit. For each pool, 8 x 25 µL reactions were performed and pooled after PCR cleanup to minimize jackpot effects. A second round of PCR attached Nextera XT barcodes according to **Table S4** using the “Round 2” reaction recipe and cycling conditions in **Table S5** and **Table S6** with the pooled Round 1 reactions as templates.

*High-Throughput Sequencing Data Processing*

The code for data processing using the Linux command-line interface (bash) is given following each explanation. Data were trimmed using Trimmomatic[1] with a 2-unit sliding quality window of 30 and a minimum length of 30. Sequences were cropped to 243 bp.

*java -jar trimmomatic-0.36.jar SE input_forward_HTS001.fastq.gz HTS001_trimmed SLIDINGWINDOW:2:30 MINLEN:30 CROP:243*

Reads were then aligned to the wild-type PrgI reference gene with Burrows–Wheeler Aligner (BWA-MEM)[2] and piped into Samtools[3] to convert to a bam file.

*bwa mem -p Reference/ref.fasta HTS001_trimmed.fastq | samtools view -bT Reference/ref.fasta -o HTS001.bam*

Reads that fully mapped to PrgI were kept for further analysis.

*samtools view HTS001.bam | grep “243M” | sort | less -S>HTS001.txt*

The trimmed reads were further processed to generate a secretion fitness landscape using code written in-house (see below for details).

Secretion Titer Score Definitions

$m$: one of the 20 canonical amino acids.

$P_{i}$: pool of screened clones sorted by secretion titer relative to PrgI^WT^. Nonfunctional clones are in pool $A$, and pools $B-J$ contain functional clones with relative secretion titer increasing from $B$ to $J$.

$$P= \left[ A, B, C,D,E,F,G,H,I,J \right]$$

$s_{P_{i}}$ : a secretion titer score assigned to each pool $P_{i}$.

$$s_{P}= \left[ -1, 1, 2, 3, 4, 5, 6, 7, 8, 9 \right]$$

$A_{P_{i},p,m}$ : an abundance score in pool $P_{i}$, indexed by position $p$ and mutation $m$.

$B_{P_{i},p,m}$: a binary array recording presence (“1”) or absence (“0”) of a mutation $m$ at position $p$ in pool $P_{i}$.

${PA}_{P_{i},p,m}$ : a percent abundance score for each mutation $m$ at position $p$ in pool $P_{i}$.

$S_{P_{i},p,m}$ : a secretion titer score for each mutation $m$ at position $p$ in pool $P_{i}$.

${SS}_{p,m}$: an array of secretion titer scores for mutation $m$ at position $p$.

${PAW}_{p,m}$: an array of percent abundances for mutation $m$ at position $p$.

$w_{p,m}$: an array of weighted percent abundances for mutation $m$ at position $p$.

${WS}_{p,m}$ : a weighted average secretion titer score for each mutation $m$ at position $p$ in pool $P_{i}$.

$\bar{{WS}_{p}}$ : average secretion titer score per residue

Secretion Titer Score Calculations

Sequences were trimmed and aligned using code written in-house. Following the data processing described above, a text file was produced containing one sequencing read per line. The text file was read into Python, and only lines starting with “ATG” and ending with “TAA” were kept.

A second quality control step was implemented in Python: sequences with read counts < 20, sequences that matched the wild-type PrgI sequence, and sequences that contained more than one mutation were discarded.

Sequences that survived the more stringent quality control step were translated, and two matrices were populated: an abundance array $A$ with read counts for each mutation at each position, and a binary array $B$ containing a “1” for a mutation present at a position and a “0” for a mutation not present at a position.

Percent abundance for each mutation at each position was generated by dividing by the total number of read counts:

$${PA}_{P_{i},p,m}= \frac{A_{P_{i},p,m}}{\sum A_{P_{i},p,m}}$$

A secretion titer score was assigned for $B_{P_{i},p,m}=1$ to generate a new matrix for each pool with the appropriate secretion titer score at mutations that were present in that pool:

$$S_{P_{i},p,m}= B_{P_{i},p,m}*s_{P_{i}}$$

A weighted average secretion score was calculated using ${PA}_{P_{i},p,m}$ to compensate for the appearance of mutations in multiple pools. Secretion titer scores and percent abundances from all pools were collected into arrays for each mutation $m$ at position $p:$

$${{SS}_{p,m}}_{i}= S_{P_{i},p,m}$$

$${{PAW}_{p,m}}_{i}={PA}_{P_{i}, p,m}$$

An array of weights was generated from the percent abundances and multiplied by the list of secretion scores to calculate a weighted average secretion titer score:

$${w_{p,m}}_{i}=\frac{{{PAW}_{p,m}}_{i}}{\sum{{PAW}_{p,m}}_{i}}$$

$${WS}_{p,m}={SS}_{p,m}\cdot{w_{p,m}}^{T}$$

${WS}_{p,m}$ is plotted in **Fig 4.** The average secretion score per residue was calculated by averaging ${WS}_{p,m}$ for each $p$, ignoring missing values:

$$\bar{{WS}_{p}}=\frac{\sum{WS}_{p,m}}{19-\sum{(\# NaN)}_{p}}$$

**Supplementary Tables**

| Strain | Genotype | Reference |
| --- | --- | --- |
| ASTE13 | LT2-derived lab strain similar to DW01 | This study; DW01 [4] |
| ASTE13 *∆prgI* | *∆prgI* | [5] |
| ASTE13 *prgI::catsacB* | *prgI::catsacB* | [6] |
| DTE509 | ASTE13 *prgI::prgI^L9A^* | [6] |
| DTE510 | ASTE13 *prgI::prgI^Q48A^* | [6] |
| DTE511 | ASTE13 *prgI::prgI^Y54A^* | [6] |
| DTE512 | ASTE13 *prgI::prgI^D70A^* | [6] |
| DTE513 | ASTE13 *prgI::prgI^P41A^* | [6] |
| DTE514 | ASTE13 *prgI::prgI^P41C^* | This study |
| DTE515 | ASTE13 *prgI::prgI^P41D^* | This study |
| DTE516 | ASTE13 *prgI::prgI^P41E^* | This study |
| DTE517 | ASTE13 *prgI::prgI^P41F^* | This study |
| DTE518 | ASTE13 *prgI::prgI^P41G^* | This study |
| DTE519 | ASTE13 *prgI::prgI^P41H^* | This study |
| DTE520 | ASTE13 *prgI::prgI^P41I^* | This study |
| DTE521 | ASTE13 *prgI::prgI^P41K^* | This study |
| DTE522 | ASTE13 *prgI::prgI^P41L^* | This study |
| DTE523 | ASTE13 *prgI::prgI^P41M^* | This study |
| DTE524 | ASTE13 *prgI::prgI^P41N^* | This study |
| DTE525 | ASTE13 *prgI::prgI^P41Q^* | This study |
| DTE526 | ASTE13 *prgI::prgI^P41R^* | This study |
| DTE527 | ASTE13 *prgI::prgI^P41S^* | This study |
| DTE528 | ASTE13 *prgI::prgI^P41T^* | This study |
| DTE529 | ASTE13 *prgI::prgI^P41V^* | This study |
| DTE530 | ASTE13 *prgI::prgI^P41W^* | This study |
| DTE531 | ASTE13 *prgI::prgI^P41Y^* | This study |
| sLAB190 | ASTE13 *sptP::sptP^(1-167)^-phoA-2xFLAG-6xHis* | This study |
| sLAB191 | ASTE13 *sptP::sptP^(1-167)^-phoA-2xFLAG-6xHis prgI::catsacB* | This study |
| sLAB192 | ASTE13 *sptP::sptP^(1-167)^-phoA-2xFLAG-6xHis prgI::prgI^P41A^* | This study |
| sLAB193 | ASTE13 *sptP::sptP^(1-167)^-phoA-2xFLAG-6xHis prgI::prgI^P41C^* | This study |
| sLAB194 | ASTE13 *sptP::sptP^(1-167)^-phoA-2xFLAG-6xHis prgI::prgI^P41M^* | This study |
| sLAB195 | ASTE13 *sptP::sptP^(1-167)^-phoA-2xFLAG-6xHis prgI::prgI^P41T^* | This study |
| sLAB196 | ASTE13 *sptP::sptP^(1-167)^-phoA-2xFLAG-6xHis prgI::prgI^P41V^* | This study |
| sLAB203 | ASTE13 *sptP::sptP^(1-167)^-phoA-2xFLAG-6xHis prgI::prgI^P41D^* | This study |
| sLAB204 | ASTE13 *sptP::sptP^(1-167)^-phoA-2xFLAG-6xHis prgI::prgI^P41E^* | This study |
| sLAB205 | ASTE13 *sptP::sptP^(1-167)^-phoA-2xFLAG-6xHis prgI::prgI^P41F^* | This study |
| sLAB206 | ASTE13 *sptP::sptP^(1-167)^-phoA-2xFLAG-6xHis prgI::prgI^P41G^* | This study |
| sLAB207 | ASTE13 *sptP::sptP^(1-167)^-phoA-2xFLAG-6xHis prgI::prgI^P41H^* | This study |
| sLAB208 | ASTE13 *sptP::sptP^(1-167)^-phoA-2xFLAG-6xHis prgI::prgI^P41I^* | This study |
| sLAB209 | ASTE13 *sptP::sptP^(1-167)^-phoA-2xFLAG-6xHis prgI::prgI^P41K^* | This study |
| sLAB210 | ASTE13 *sptP::sptP^(1-167)^-phoA-2xFLAG-6xHis prgI::prgI^P41L^* | This study |
| sLAB211 | ASTE13 *sptP::sptP^(1-167)^-phoA-2xFLAG-6xHis prgI::prgI^P41N^* | This study |
| sLAB212 | ASTE13 *sptP::sptP^(1-167)^-phoA-2xFLAG-6xHis prgI::prgI^P41Q^* | This study |
| sLAB213 | ASTE13 *sptP::sptP^(1-167)^-phoA-2xFLAG-6xHis prgI::prgI^P41R^* | This study |
| sLAB214 | ASTE13 *sptP::sptP^(1-167)^-phoA-2xFLAG-6xHis prgI::prgI^P41S^* | This study |
| sLAB215 | ASTE13 *sptP::sptP^(1-167)^-phoA-2xFLAG-6xHis prgI::prgI^P41W^* | This study |
| sLAB216 | ASTE13 *sptP::sptP^(1-167)^-phoA-2xFLAG-6xHis prgI::prgI^P41Y^* | This study |
| sLAB305 | ASTE13 *sptP::sptP^(1-167)^-phoA-2xFLAG-6xHis ∆invA* | This study |

**Table S1 – Strains used in this study.**

| Plasmid Name | ORFs under inducible control | | ORI | ab^R^ | Reference |
| --- | --- | --- | --- | --- | --- |
| P*_sic_ DH* | *sicP* | *sptP-DH-2xFLAG-6xHis* | colE1 | cam | [5] |
| P*_sic_ AP* | *sicP* | *sptP-phoA-2xFLAG-6xHis* | colE1 | cam | [7] |
| P*_lacUV5_ hilA* | *hilA* | | p15a | kan | [5] |
| *pSIM6* | *gam, beta, exo* | | pSC101 | cb | [8] |
| P*_lacUV5_ P41A* | *prgI^P41A^* | | colE1 | kan | [6] |
| P*_lacUV5_ P41C* | *prgI^P41C^* | | colE1 | kan | This study |
| P*_lacUV5_ P41D* | *prgI^P41D^* | | colE1 | kan | This study |
| P*_lacUV5_ P41E* | *prgI^P41E^* | | colE1 | kan | This study |
| P*_lacUV5_ P41F* | *prgI^P41F^* | | colE1 | kan | This study |
| P*_lacUV5_ P41G* | *prgI^P41G^* | | colE1 | kan | This study |
| P*_lacUV5_ P41H* | *prgI^P41H^* | | colE1 | kan | This study |
| P*_lacUV5_ P41I* | *prgI^P41I^* | | colE1 | kan | This study |
| P*_lacUV5_ P41K* | *prgI^P41K^* | | colE1 | kan | This study |
| P*_lacUV5_ P41L* | *prgI^P41L^* | | colE1 | kan | This study |
| P*_lacUV5_ P41M* | *prgI^P41M^* | | colE1 | kan | This study |
| P*_lacUV5_ P41N* | *prgI^P41N^* | | colE1 | kan | This study |
| P*_lacUV5_ P41Q* | *prgI^P41Q^* | | colE1 | kan | This study |
| P*_lacUV5_ P41R* | *prgI^P41R^* | | colE1 | kan | This study |
| P*_lacUV5_ P41S* | *prgI^P41S^* | | colE1 | kan | This study |
| P*_lacUV5_ P41T* | *prgI^P41T^* | | colE1 | kan | This study |
| P*_lacUV5_ P41V* | *prgI^P41V^* | | colE1 | kan | This study |
| P*_lacUV5_ P41W* | *prgI^P41W^* | | colE1 | kan | This study |
| P*_lacUV5_ P41Y* | *prgI^P41Y^* | | colE1 | kan | This study |

**Table S2 – Plasmids used in this study.**

| Sequence | Amplicon | Used to Construct |
| --- | --- | --- |
| GCAGCAAAACCCTCCGATTGTGCGCTACTGGCGGCGTATC | *prgI^P41C^* QC | P*_lacUV5_ P41C* |
| GATACGCCGCCAGTAGCGCACAATCGGAGGGTTTTGCTGC | *prgI^P41C^* QC | P*_lacUV5_ P41C* |
| GCAGCAAAACCCTCCGATGATCGCTACTGGCGGCGTATC | *prgI^P41D^* QC | P*_lacUV5_ P41D* |
| GATACGCCGCCAGTAGCGCATCATCGGAGGGTTTTGCTGC | *prgI^P41D^* QC | P*_lacUV5_ P41D* |
| GCAGCAAAACCCTCCGATGAAGCGCTACTGGCGGCGTATC | *prgI^P41E^* QC | P*_lacUV5_ P41E* |
| GATACGCCGCCAGTAGCGCTTCATCGGAGGGTTTTGCTGC | *prgI^P41E^* QC | P*_lacUV5_ P41E* |
| CAAAACCCTCCGATTTTGCGCTACTGGCGGCG | *prgI^P41F^* QC | P*_lacUV5_ P41F* |
| CGCCGCCAGTAGCGCAAAATCGGAGGGTTTTG | *prgI^P41F^* QC | P*_lacUV5_ P41F* |
| CAAAACCCTCCGATGGAGCGCTACTGGCGGC | *prgI^P41G^* QC | P*_lacUV5_ P41G* |
| GCCGCCAGTAGCGCTCCATCGGAGGGTTTTG | *prgI^P41G^* QC | P*_lacUV5_ P41G* |
| CAGCAAAACCCTCCGATCATGCGCTACTGGCGGCGTATC | *prgI^P41H^* QC | P*_lacUV5_ P41H* |
| GATACGCCGCCAGTAGCGCATGATCGGAGGGTTTTGCTG | *prgI^P41H^* QC | P*_lacUV5_ P41H* |
| CAAAACCCTCCGATATAGCGCTACTGGCGGCG | *prgI^P41I^* QC | P*_lacUV5_ P41I* |
| CGCCGCCAGTAGCGCTATATCGGAGGGTTTTG | *prgI^P41I^* QC | P*_lacUV5_ P41I* |
| CAAAACCCTCCGATAAGGCGCTACTGGCG | *prgI^P41K^* QC | P*_lacUV5_ P41K* |
| CGCCAGTAGCGCCTTATCGGAGGGTTTTG | *prgI^P41K^* QC | P*_lacUV5_ P41K* |
| CAAAACCCTCCGATTTGGCGCTACTGGCG | *prgI^P41L^* QC | P*_lacUV5_ P41L* |
| CGCCAGTAGCGCCAAATCGGAGGGTTTTG | *prgI^P41L^* QC | P*_lacUV5_ P41L* |
| CAAAACCCTCCGATATGGCGCTACTGGCGGCG | *prgI^P41M^* QC | P*_lacUV5_ P41M* |
| CGCCGCCAGTAGCGCCATATCGGAGGGTTTTG | *prgI^P41M^* QC | P*_lacUV5_ P41M* |
| CAAAACCCTCCGATAATGCGCTACTGGCGGCG | *prgI^P41N^* QC | P*_lacUV5_ P41N* |
| CGCCGCCAGTAGCGCATTATCGGAGGGTTTTG | *prgI^P41N^* QC | P*_lacUV5_ P41N* |
| CAGCAAAACCCTCCGATCAAGCGCTACTGGCGGCGTATC | *prgI^P41Q^* QC | P*_lacUV5_ P41Q* |
| GATACGCCGCCAGTAGCGCTTGATCGGAGGGTTTTGCTG | *prgI^P41Q^* QC | P*_lacUV5_ P41Q* |
| GCAGCAAAACCCTCCGATAGAGCGCTACTGGCGGCGTATC | *prgI^P41R^* QC | P*_lacUV5_ P41R* |
| GATACGCCGCCAGTAGCGCTCTATCGGAGGGTTTTGCTGC | *prgI^P41R^* QC | P*_lacUV5_ P41R* |
| GCAGCAAAACCCTCCGATAGTGCGCTACTGGCGGCGTATC | *prgI^P41S^* QC | P*_lacUV5_ P41S* |
| GATACGCCGCCAGTAGCGCACTATCGGAGGGTTTTGCTGC | *prgI^P41S^* QC | P*_lacUV5_ P41S* |
| GCAGCAAAACCCTCCGATACAGCGCTACTGGCGGCGTATC | *prgI^P41T^* QC | P*_lacUV5_ P41T* |
| GATACGCCGCCAGTAGCGCTGTATCGGAGGGTTTTGCTGC | *prgI^P41T^* QC | P*_lacUV5_ P41T* |
| GCAGCAAAACCCTCCGATGTAGCGCTACTGGCGGCGTATC | *prgI^P41V^* QC | P*_lacUV5_ P41V* |
| GATACGCCGCCAGTAGCGCTACATCGGAGGGTTTTGCTGC | *prgI^P41V^* QC | P*_lacUV5_ P41V* |
| CAAAACCCTCCGATTGGGCGCTACTGGCGGCG | *prgI^P41W^* QC | P*_lacUV5_ P41W* |
| CGCCGCCAGTAGCGCCCAATCGGAGGGTTTTG | *prgI^P41W^* QC | P*_lacUV5_ P41W* |
| CAAAACCCTCCGATTATGCGCTACTGGCGGCG | *prgI^P41Y^* QC | P*_lacUV5_ P41Y* |
| CGCCGCCAGTAGCGCATAATCGGAGGGTTTTG | *prgI^P41Y^* QC | P*_lacUV5_ P41Y* |
| AACATACTGCAGGAATATGCTAAAGTATGAGGAGAGAAAA tgtgacggaagatcacttcg | *cat-sacB* | ASTE13 *sptP::catsacB* |
| GCTTACTTTCAGATAGTTCTAAAAGTAAGCTATGTTTTTA atcaaagggaaaactgtccatat | *cat-sacB* | ASTE13 *sptP::catsacB* |
| CTTGAGTCATTTGTGAATCAGCAGGAAGCGCTCAAAAACATACTGCAGGAATATGCTAAAGTATGAGGAGAGAAAA ttgaataatttaacgttgtcttcg | *sptP^(1-167)^-phoA-2xFLAG-6xHis* | ASTE13 *sptP::sptP^(1-167)^-phoA-2xFLAG-6xHis* |
| ACTTTCTATCGCGGCAAACAAATAATTATACAGAAATAGCTTACTTTCAGATAGTTCTAAAAGTAAGCTATGTTTTTA ttagtggtgatggtgatgatgc | *sptP^(1-167)^-phoA-2xFLAG-6xHis* | ASTE13 *sptP::sptP^(1-167)^-phoA-2xFLAG-6xHis* |
| CCCAAGCCCACTTTAATTTAACGTAAATAAGGAAGTCATT atggcaacaccttggtcagg | *prgI* | All ASTE13 *prgI* variants |
| GGACAATAGTTGCAATCGACATAATCCACCTTATAACTGA ttaacggaagttctgaataatggc | *prgI* | All ASTE13 *prgI* variants |
| CTATAGTGCTGCTTTCTCTACTTAACAGTGCTCGTTTACG tgtgacggaagatcacttcg | *cat-sacB* | ASTE13 *sptP::sptP^(1-167)^-phoA-2xFLAG-6xHis ∆invA* |
| GCCCTTATATTGTTTTTATAACATTCACTGACTTGCTAT atcaaagggaaaactgtccat | *cat-sacB* | ASTE13 *sptP::sptP^(1-167)^-phoA-2xFLAG-6xHis ∆invA* |
| TTATATTGTTTTTATAACATTCACTGACTTGCTATCGTAAACGAGCACTGTTAAGTAGAGAAAGCAGCAC | N/A | ASTE13 *sptP::sptP^(1-167)^-phoA-2xFLAG-6xHis ∆invA* |
| TCGTCGGCAGCGTCAGATGTGTATAAGAGACAG atggcaacaccttggtcag | *prgI* | PrgI library for NGS – PCR step 1 |
| GTCTCGTGGGCTCGGAGATGTGTATAAGAGACAG ttaacggaagttctgaataatggc | *prgI* | PrgI library for NGS – PCR step 1 |

**Table S3 – Primers used in this study.**

| Pool | Relative Secretion Titer | Number of Clones | Sample Volume  for Mixture (μL) | Nextera XT  Primer i5 | Nextera XT  Primer i7 |
| --- | --- | --- | --- | --- | --- |
| A | 0.01-0.6 | 2015 | 5 | N707 | S502 |
| B | 0.6-0.8 | 575 | 10 | N710 | S502 |
| C | 0.8-1.0 | 691 | 10 | N711 | S502 |
| D | 1.0-1.2 | 480 | 20 | N712 | S502 |
| E | 1.2-1.4 | 236 | 20 | N714 | S502 |
| F | 1.4-1.6 | 151 | 20 | N705 | S503 |
| G | 1.6-1.8 | 77 | 30 | N706 | S503 |
| H | 1.8-2.0 | 75 | 30 | N707 | S503 |
| I | 2.0-2.5 | 70 | 30 | N710 | S503 |
| J | >2.5 | 36 | 50 | N711 | S503 |

**Table S4 – Pools for high throughput sequencing according to relative secretion titer.**

| Component | Round 1 (25 µL x 8 per pool) | Round 2 (50 µL x 1 per pool) |
| --- | --- | --- |
| 5X HF Buffer (NEB) | 5 µL | 10 µL |
| 10 mM dNTPs (NEB) | 0.5 µL | 1 µL |
| 10 µM FWD primer | 1.25 µL | 2.5 µL |
| 10 µM REV primer | 1.25 µL | 2.5 µL |
| Template DNA | 2.5 µL of 5 ng/µL gDNA | 5 µL purified and combined Round 1 reaction |
| Phusion (NEB) | 0.25 µL | 0.5 µL |
| H_2_O | 14.25 µL | 28.5 µL |

**Table S5 – PCR reactions for high throughput sequencing library preparation.**

| Step | Round 1 (25 µL x 8 per pool)  T (°C) Time (sec) | Round 2 (50 µL x 1 per pool)  T (°C) Time (sec) |
| --- | --- | --- |
| Initial Denaturation | 98 60 | 98 30 |
| Amplification | 98 10 | 98 10 |
| (Round 1 – 22 cycles) | 62 15 | 61 15 |
| (Round 2 – 8 cycles) | 72 30 | 72 30 |
| Elongation | 72 300 | 72 300 |
| Hold | 4 indefinite | 4 indefinite |

**Table S6 – PCR cycling conditions for high throughput sequencing library preparation.**

**Supplementary references**

[1] Bolger, A. M., Lohse, M. & Usadel, B. Trimmomatic: a flexible trimmer for Illumina sequence data. Bioinformatics 30, 2114–2120 (2014).

[2] Li, H. & Durbin, R. Fast and accurate short read alignment with Burrows-Wheeler transform. Bioinformatics 25, 1754–1760 (2009).

[3] Li, H. et al. The sequence alignment/map format and SAMtools. Bioinformatics 25, 2078–2079 (2009).

[4] Song M, Sukovich DJ, Ciccarelli L, Mayr J, Fernandez-Rodriguez J, Mirsky EA, et al. Control of type III protein secretion using a minimal genetic system. Nature Communications. 2017;8:14737.

[5] Metcalf KJ, Finnerty C, Azam A, Valdivia E, Tullman-Ercek D. Using Transcriptional Control To Increase Titers of Secreted Heterologous Proteins by the Type III Secretion System. Applied and Environmental Microbiology. 2014;80:5927–34.

[6] Metcalf KJ. Engineering heterologous protein secretion for improved production. University of California, Berkeley; 2016.

[7] Metcalf KJ, Bevington JL, Rosales SL, Burdette LA, Valdivia E, Tullman-Ercek D. Proteins adopt functionally active conformations after type III secretion. Microbial Cell Factories. 2016;15:213.

[8] Thomason LC, Sawitzke JA, Li X, Costantino N, Court DL. Recombineering: Genetic Engineering in Bacteria Using Homologous Recombination. Current Protocols in Molecular Biology. 2014;106:1.16.1-1.16.39.
